# Quantitative Genetic Analyses of Male Color Pattern and Female Mate Choice in a Pair of Cichlid Fishes of Lake Malawi, East Africa

**DOI:** 10.1371/journal.pone.0114798

**Published:** 2014-12-10

**Authors:** Baoqing Ding, Daniel W. Daugherty, Martin Husemann, Ming Chen, Aimee E. Howe, Patrick D. Danley

**Affiliations:** Biology Department, Baylor University, One Bear Place #97388, Waco, Texas, 76798, United States of America; Virginia Tech Virginia, United States of America

## Abstract

The traits involved in sexual selection, such as male secondary sexual characteristics and female mate choice, often co-evolve which can promote population differentiation. However, the genetic architecture of these phenotypes can influence their evolvability and thereby affect the divergence of species. The extraordinary diversity of East African cichlid fishes is often attributed to strong sexual selection and thus this system provides an excellent model to test predictions regarding the genetic architecture of sexually selected traits that contribute to reproductive isolation. In particular, theory predicts that rapid speciation is facilitated when male sexual traits and female mating preferences are controlled by a limited number of linked genes. However, few studies have examined the genetic basis of male secondary sexual traits and female mating preferences in cichlids and none have investigated the genetic architecture of both jointly. In this study, we artificially hybridized a pair of behaviorally isolated cichlid fishes from Lake Malawi and quantified both melanistic color pattern and female mate choice. We investigated the genetic architecture of both phenotypes using quantitative genetic analyses. Our results suggest that 1) many non-additively acting genetic factors influence melanistic color patterns, 2) female mate choice may be controlled by a minimum of 1–2 non-additive genetic factors, and 3) F_2_ female mate choice is not influenced by male courting effort. Furthermore, a joint analysis of color pattern and female mate choice indicates that the genes underlying these two traits are unlikely to be physically linked. These results suggest that reproductive isolation may evolve rapidly owing to the few genetic factors underlying female mate choice. Hence, female mate choice likely played an important role in the unparalleled speciation of East African cichlid fish.

## Introduction

The genetic architecture of traits experiencing sexual selection influences phenotypic evolution and speciation [1]. The pattern and rate of response of a trait to selection depends on several factors including the strength of selection, the presence of genetic variation, the mode of inheritance, genetic correlations with other traits, the numbers of genes and alleles underlying a phenotype, the distribution of allelic effects, and patterns of pleiotropy, dominance, and epistasis [1]–[4]. Despite their importance, empirical studies examining these factors within the context of sexual selection and speciation are sparse, particularly in vertebrates. Here we investigate the genetic architecture of melanistic coloration and female mate choice in the context of the diversification of East African cichlids.

The radiation of East Africa's cichlid fishes has produced the largest extant vertebrate diversification identified to date. More than 2000 cichlid species have evolved in East Africa within the past 10 million years [5]. This unparalleled vertebrate radiation is primarily concentrated in the three East African Great Lakes: Lake Tanganyika, Lake Victoria, and Lake Malawi. Within Lake Malawi, over 600 cichlid species [6] have evolved from a single common ancestor since the formation of the lake within the past one to four million years [5], [7]. A hallmark of this diversification is the extraordinary diversity of male color patterns. The diversity in color patterns of East African cichlids has long been attributed to sexual selection via female mate choice [8]–[10] suggesting that strong sexual selection significantly contributed to their speciation [11]–[15]. Although some have suggested that color patterns may have evolved in response to ecological pressures [16]–[19] or male-male competition [20]–[22], many empirical studies have documented the role that male color patterns, i.e. colored ornamentation [23], body hue [24], [25], and melanistic patterns [26]–[28], play in sexual selection via female mate choice.

Speciation via sexual selection can be greatly facilitated by the physical linkage of the genes contributing to female mate choice and the preferred male phenotype [29], [30]. In many models of the diversification of East African cichlids, incipient species chose their mates based on male color patterns [10], [31], [32]. This targeted mate selection impedes gene flow and contributes to reproductive isolation [33]–[36]. Speciation can be further expedited when the genes underlying male secondary sexual traits and female mate choice are physically linked. Tight physical linkage can reduce the likelihood of recombination events, which can lead to the accumulation of genetic incompatibilities, and facilitate speciation [10], [29], [31], [37]–[41].

Despite their importance, the genetic mechanisms contributing to male coloration and female mate choice involved in cichlid diversification remain poorly understood. The genetics of male color pattern in cichlids are relatively well studied [11]–[13], [42] and several independent studies suggest that male coloration [43]–[45] is controlled by a small number of genes. However, much less is known about the genetics of female mate choice in this system. One study suggests that female mate choice is influenced by a small number of genes [29]. Additional studies in this or other systems are rare since accurate measurements of female mate choice are difficult to obtain and are time consuming [46], [47]. As a result, even fewer studies have performed joint genetic analyses of male sexual traits and female mate choice [48].

To examine the quantitative genetic basis of male color pattern and female mate choice, we employed a well-established method to quantify color pattern [49] and designed a novel assay to test female mate choice in cichlids. We performed quantitative genetic analyses on both phenotypes to understand the mode of gene action and number of loci involved in these evolutionary significant traits. Further, we tested for physical linkage between color pattern and female mate choice. Specifically, we hypothesized that if the genes underlying color pattern and female mate choice were physically linked, the phenotypes would co-segregate in the hybrid F_2_ generation. If such linkage is observed, it may provide insights into the mechanisms facilitating the rapid divergence of cichlid species [30].

## Materials and Methods

### Focal species and their hybrids

**Figure 1:**
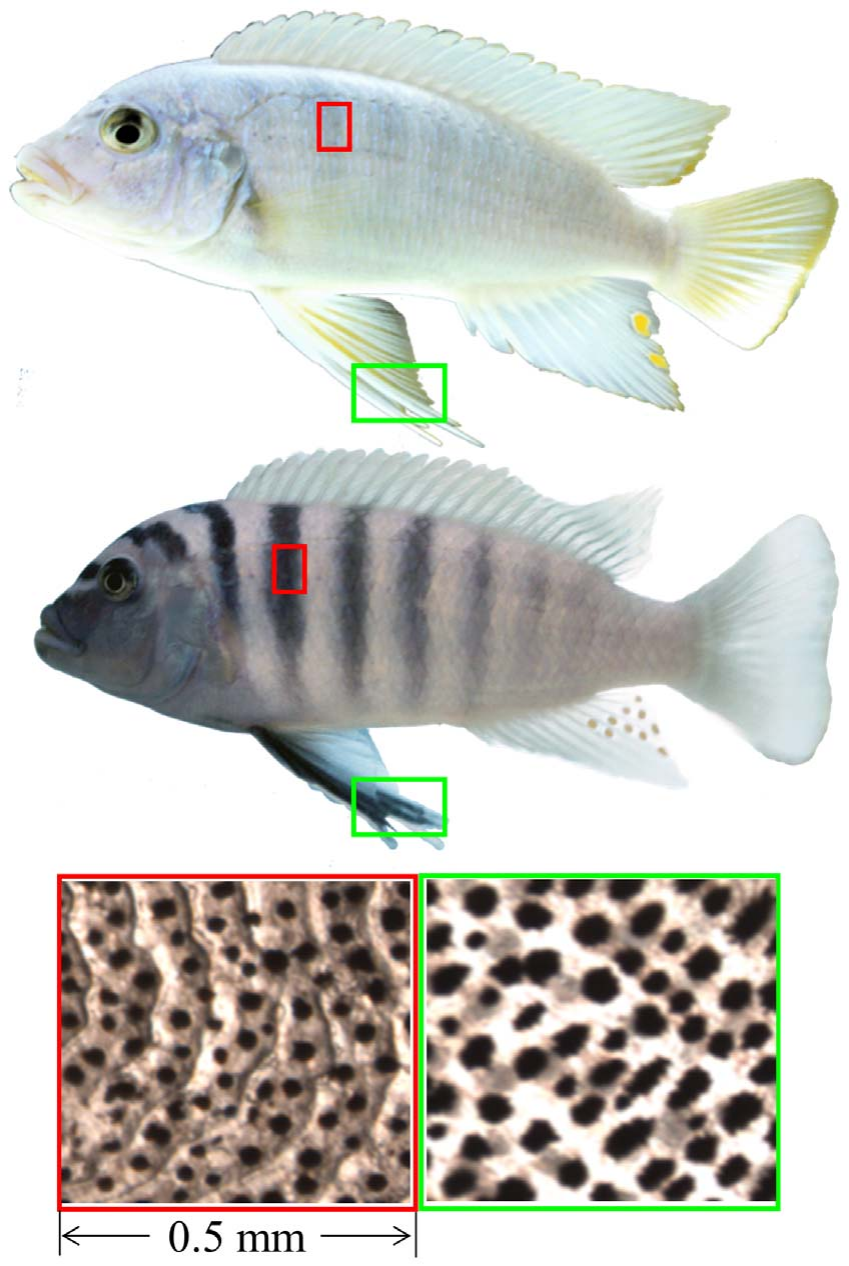
Male individuals of both species with indication where scale and fin samples were taken. *Maylandia benetos* is at the top panel, *Maylandia zebra* is at the middle panel, and scale and fin tissue samples are at the lower panel. The red box indicates the area where the scale samples for melanophore counts were taken, the green box indicates where the melanophores in fins were counted.

The Baylor University Institutional Animal Care and Use Committee approved this research. The protocol number is 08–09. Lake Malawi National Park, the Malawi Fisheries Department granted our sampling permit.*Maylandia zebra* and *Maylandia benetos* are a pair of sympatric rock-dwelling cichlid species from Lake Malawi. While *M. zebra* is a cosmopolitan species that occurs at many locations across the lake, *M. benetos* only inhabits Mazinzi Reef in the southeastern arm of the lake [18]. At Mazinzi Reef, these two species coexist in sympatry but do not hybridize: in over 400 hours of observation, no interspecific courtship or intermediate hybrid individuals were observed (Danley, pers. obs.). The two study species differ primarily in their melanistic markings [28]; *M. zebra* has a bright blue ground color with 5–7 dark body bars, a black cheek, and dark banding on the pelvic fin, whereas *M. benetos* has a bright blue ground color and faint body barring and pelvic fin markings. Although territorial males of both species maintain a bright blue hue, the melanisitic markings of *M. zebra* become more conspicuous when the males are territorial and fade when the male is stressed (Fig. 1). In contrast the melanistic markings of *M. benetos* become faint when territorial and their melanistic markings only become apparent when stressed. Females and subdominant males of both species are drab brown (*M. zebra*) or drab olive (*M. benetos*). Female *M. zebra* display similar melanisitic markings as observed in male *M. zebra*; however, the markings are much less conspicuous than those of the territorial *M. zebra* males [28]. A previous study of this species pair found that visual cues alone are sufficient for conspecific mate recognition and that melanistic patterning may play a significant role in the reproductive isolation of these species [28]. The mating behaviors of the two species are nearly identical [50] and involve elements such as quiver, lateral display and circling. Both species mate year round, are maternal mouthbrooders, and have a reproductive cycle that varies from 28 to 30 days at 28°C under laboratory conditions (Ding, pers. obs.).

Since the two species do not hybridize naturally, hybrid F_1_ offspring were obtained by artificial fertilization. Mature eggs from *M. benetos* and sperm from *M. zebra* were obtained by gently compressing the abdomen of the fish. The gametes were mixed for five minutes in a petri dish (136 mm diameter ×14 mm deep) filled with tank water and the fertilized eggs were transferred into a glass incubator where they were incubated at 28°C until the larvae could swim independently. The reciprocal F_1_ cross, *M. zebra* (♀)×*M. benetos* (♂), was created with the same method. F_2_ hybrids were obtained by intercrossing the F_1_ individuals derived from the cross between *M. zebra* (♀)×*M. benetos* (♂). The 11 *M. benetos* (♀)×*M. zebra* (♂) F_1_ broods produced only F_1_ females thus no F_2_ hybrids could be obtained from this direction. Backcrosses were generated by crossing *M. zebra* (♀)×*M. benetos* (♂) F_1_ hybrids to the two parental species. Prior to the F_2_ reaching sexual maturity, male and female F_2_ hybrids were isolated to avoid any sexual experience. Both parental stocks were reared under laboratory conditions for at least 10 generations. Water temperature was kept between 26° and 28°C. Light was kept at a 12-hour day/night cycle using timer controlled fluorescent lights. Fish were fed a mixture of food flakes twice daily.

### Quantification of color pattern

Fish were allowed to grow for a minimum of one year. Scales and pelvic fins were collected to quantify the differences in color pattern between the two species. Both males and females were sampled to compare the color pattern within each species and hybrid brood. Scales from the first complete body bar (one scale from immediately below the lateral line) and the distal third of the pelvic fin were collected (Fig. 1). Scale and pelvic fin samples were then emerged in K^+^ rich aggregating fluid to contract melanophores [49]. Melanophore quantity was assessed by counting the number of melanophores which occur in a 0.25 mm^2^ area from both tissues at 30x magnification through a Nikon stereo microscope (SMZ1500).

### Phenotyping of female mate choice

**Figure 2:**
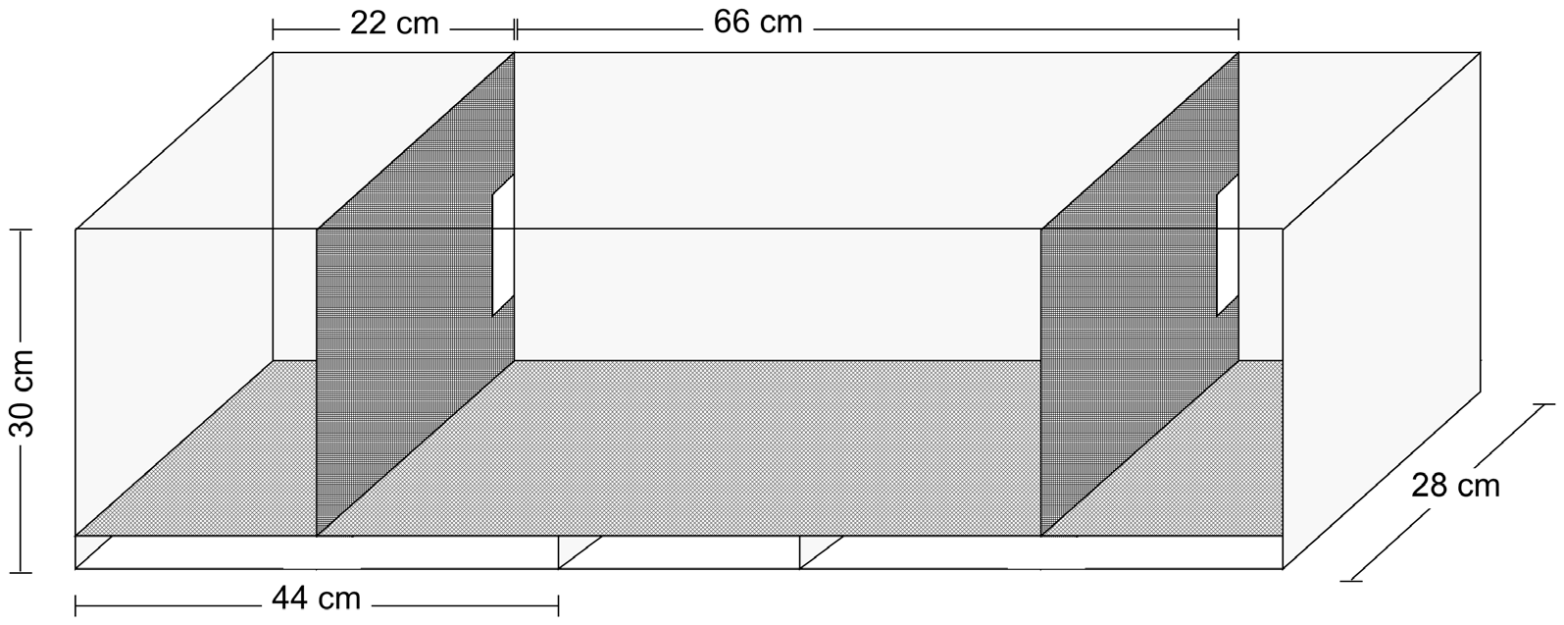
Illustration of experimental mate choice assay tank design. Males of the two species were placed in two side partitions, while trial female were placed in the center compartment of the tank.

Mate choice experiments were performed in 110 cm×28 cm×30 cm tanks split into three compartments: two male compartments that flanked a central female compartment (Fig. 2). Plastic grating composed of 15 mm squares (‘egg crate’) was used to divide the compartments and produce a false bottom to the tank. The false bottom prevented females from collecting their eggs during mating. Each divider separating the three compartments contained an opening sufficiently large to allow free movement of the test females between compartments, but small enough to prevent the males from escaping their respective areas. A male *M. zebra* was randomly placed in one flanking compartment and a male *M. benetos* was placed in the opposing flanking compartment. Both males were allowed to acclimate for at least 24 hours. In all experimental trials, males were size-matched for standard length (<2 mm difference). The side allocated to the male of each species was randomized to control for side bias. Ten size-matched pairs of males were used in this experiment.

Making accurate measurements of female mate choice can be challenging and time consuming [46], [47]. Several methods have been developed to quantify cichlid fish mate choice including (listed in order of increasing measurement confidence [51]: time in association with a male [27], behavioral assays [29] and egg counts [52]). Because egg counts are a direct rather than a proxy measure of female mate choice, this was the primary metric of female mate choice used in our experiment. The amount of time a female spent in association with each male in their respective compartments was recorded to evaluate the reliability of association time as an indicator of female mate choice. In addition, male courting effort was quantified to estimate its effects on female mate choice.

In each mate choice trial, a single reproductive female, as determined by its swollen abdomen and slightly protruded genital papilla, was introduced to the central compartment. The behaviors of both males and the female were video recorded for two hours after the introduction of the female to the trial arena. After reviewing the video and verifying that female had visited both male compartments, a 20 minute video clip was used to quantify male courting effort. Within the 20 minute video, three elements of male courting behavior were scored: quiver, lateral display, and circling. While quivering, a male swiftly swims toward a female, exaggeratedly beats its tail, and flexes its body along its long axis. During a lateral display, the male erects its fins and orients parallel or perpendicular to the female in the water column above its territory. If the female does not break off the courtship event, the pair will begin circling which occurs when the male and female swim in tight circles over the spawning area [53]. Thirty randomly selected F_2_ female mate choice behavior videos were scored to evaluate the effect of male courtship effort on F_2_ female mate choice. The number of times each male performed a quiver, lateral display, or circling behavior was used to quantify male courting effort (S1 Video. Female mate choice assay tank).

Female mate choice was quantified based on patterns of egg laying. The number of eggs laid beneath each of the three compartments (*M. zebra* compartment, *M. benetos* compartment, and/or the central compartment that lacked a stimulus male) was recorded; trials were discarded if eggs were laid in the center partition. Females typically laid eggs within 24 hours of their introduction to the test tank. Two hundred thirty one females were evaluated for their mate choice. Among these, 10 females from each parental species were tested once, whereas 29 F_1_ and 182 F_2_ females were each tested three times against three different pairs of males in three different arenas. The mate choice of the backcrosses was not examined.

### Statistical analyses

To compare male and female color pattern differences in scale and pelvic fin melanophore counts of different generations, a two-factor analysis of variance (ANOVA) was performed.

We used quantitative genetic methods to investigate the genetic architecture of color pattern and female mate choice. In order to investigate the mode of gene action for melanisitic color pattern, we compared the additive and the additive-dominance model using the joint-scaling test [54]. Tests for epistasis on melanistic color pattern were conducted following Lynch and Walsh [55]. Using this method, the test statistic, Δ, is calculated as the difference in means between the F_2_ and the weighted means of the parentals and F_1_. The joint scaling test and tests for epistasis were not used to investigate the mode of gene action for female mate choice as female mate choice in the parentals was not normally distributed (specifically, the parental variances equaled 0). The number of genetic factors underlying both coloration phenotypes and female mate choice were estimated using the Castle-Wright estimator following Lynch and Walsh [55]. Briefly, using the means and variances of the parental, F_1_ and F_2_ phenotypic distributions, the number of segregating genetic factors that are likely to be responsible for the quantitative differences of the traits can be inferred. These factors were then adjusted using Zeng's correction [56]. Assuming an additive genetic effect on female mate choice, we expect the mean of female mate choice in the reciprocal F_1_ hybrids and F_2_ to be 0. We tested this hypothesis with a one sample *t*-test.

Female mate choice was quantified using a mate choice index expressed as the number of eggs laid in the *M. zebra* compartment minus the number of eggs laid in the *M. benetos* compartment, divided by the total number of eggs laid in the trial. This index ranges from +1 (females that exclusively choose *M. zebra* males) to −1 (females that exclusively choose *M. benetos* males). The average of the mate choice index was used as an overall estimate of F_1_ and F_2_ female mate choice.

Following a similar method used by Haesler and Seehausen [29], male courting effort was quantified by regressing the number of male displays (lateral display, quiver, circling) in a 20 minute interval against the amount of time that the female spent in the male's compartment. The residuals of the male courting effort are independent of the time that the female was present in each male compartment.

To test whether female mate choice was influenced by male courting effort, we separately examined the effect of each male courting effort (lateral display, quiver, circling) on female mating preference: males that successfully mated would be expected to have exerted more effort than those males that were rejected. A Mann-Whitney-Wilcoxon test was used to test for this difference. This test was also performed to evaluate the time spent in association with successful and rejected males. The videos of 24 F_2_ females (10 exclusively mated with *M. zebra* and 14 exclusively mated with *M. benetos*) were selected to evaluate the effect of male courting efforts and the utility of female time in association as a predictor of female mate choice. The six females that mated with both males were excluded from this analysis.

To evaluate female maternal effects, we tested for possible differences in mate choice between the F_1_ reciprocal crosses with a Welch two sample *t*-test. To test whether the mate choice in the F_2_ female population deviates from random mating, a χ^2^ goodness of fit test was performed.

Finally, we investigated the phenotypic correlation between color pattern and female mate choice by comparing the color patterns of the F_2_ females that exclusively mated with *M. zebra* to those that exclusively mated with *M. benetos*. Only those females that mated exclusively with one species were used as these provided the most reliable estimate of mate choice. If the genes contributing to female mate choice were physically linked with the genes influencing color pattern, we would expect the co-segregation of color pattern and mate choice phenotypes in the F_2_: the F_2_ females which exclusively mated with *M. benetos* (*M. zebra*) males would resemble *M. benetos* (*M. zebra*) in their color pattern. An ANOVA was used to test for this phenotypic difference between F_2_ females which exclusively mated with either *M. benetos* or *M. zebra*. All melanophore count data were square root transformed to improve normality. The data analyses were performed in R [57].

## Results

### Color pattern general statistics

556 fish were phenotyped in both scale and fin tissues to quantify color pattern phenotypes. No statistically significant difference was found between males and females in both scale (*F* = 0.264, *d.f.* = 1, *p = *0.607) and fin melanophore counts (*F* = 0.683, *d.f.* = 1, *p = *0.409) across all lines. As a result, male and female melanophore data were pooled for subsequent analysis.

Of the 556 individuals phenotyped for color pattern, 86 (61 males, 25 females) were *M. zebra* and 47 (34 males, 13 females) were *M. benetos.* Ninety four F_1_ hybrids (48 males, 46 females) derived from five independent broods (2 *M. zebra* (♀)×*M. benetos* (♂) broods with 17 individuals and 3 *M. benetos* (♀)×*M. zebra* (♂) broods with 77 individuals) were phenotyped for color pattern. Two hundred seventy F_2_ individuals (62 males, 208 females) from three independent crosses were phenotyped for color pattern. A total of 60 backcross individuals were included in the color pattern analysis: 39 individuals (20 males, 19 females) were the result of an F_1_ (*M. zebra* (♀)×*M. benetos* (♂)) backcross to *M. zebra*, while 21 individuals (14 males, 7 females) were derived from F_1_ (*M. zebra* (♀)×*M. benetos* (♂)) backcross with *M. benetos*.

**Figure 3:**
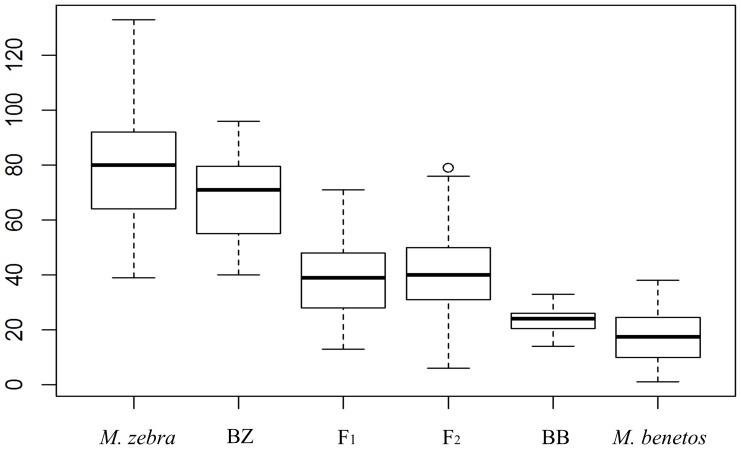
The boxplot of melanophore counts in scale of the parentals, and F_1_, F_2_ hybrids and backcrosses. BZ represents backcross with *M. zebra*, while BB represents backcross with *M. benetos*.

Scale melanophore counts varied across the broods. *Maylandia zebra* had the highest mean melanophore count (mean (SD) = 79.45 (19.92)). The *M. zebra* backcross had the next largest mean (SD) scale melanophore count at 68.56 (14.91). Both directions of the F_1_ were intermediate in their scale melanophore counts. The melanophore count for F_1_ (*M. benetos* (♀)×*M. zebra* (♂)) was 43.75 (15.68). The melanophore count for F_1_ (*M. zebra* (♀)×*M. benetos* (♂)) was 38.28 (13.44). No statistically significant differences were observed in the scale melanophore counts in the F_1_ based on the direction of the cross (t = −1.61, *d.f.* = 45.14, *p* = 0.12). The mean (SD) count of both directions of the F_1_ was 39.95 (14.29). The F_2_ melanophore scale counts were intermediate as well and had a mean (SD) of 41.06 (13.77) melanophores. The *M. benetos* backcross, (*M. zebra* (♀)×*M. benetos* (♂)) with *M. benetos*, had a mean (SD) scale melanophore count of 23.16 (5.19). With a mean (SD) scale melanophore count of 17.48 (9.72), *M. benetos* had the fewest number of scale melanophores (Fig. 3).

**Figure 4:**
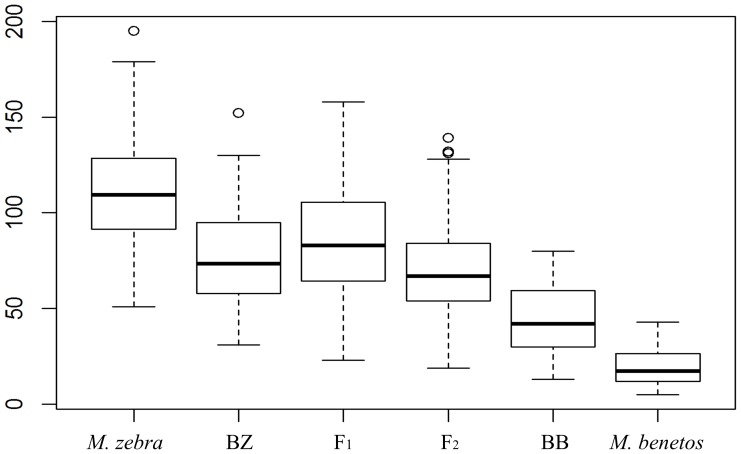
The boxplot of melanophore count in fin of the parentals, and F_1_, F_2_ hybrids and backcrosses. BZ represents backcross with *M. zebra*, while BB represents backcross with *M. benetos*.

Fin melanophore counts were distributed in a similar pattern as the scale melanophores. *Maylandia zebra* had the highest mean (SD) melanophore count (110.5 (29.51)). The backcross to *M. zebra* (F_1_ (*M. zebra* (♀)×*M. benetos* (♂)) backcross to *M. zebra*) had a mean (SD) of 77.24 (26.92) melanophores on the fins. The melanophore count for *M. benetos* (♀)×*M. zebra* (♂) F_1_ cross was 88.70 (26.55) while the melanophore count for the *M*. *zebra* (♀)×*M. benetos* (♂) F_1_ cross was 85.56 (28.97). No significant differences were observed in the fin melanophore counts in the F_1_ reciprocal crosses (t = −0.50, *d.f.* = 53.16, *p* = 0.618). The overall F_1_ hybrid mean (SD) was 86.49 (28.16) melanophores on the fin. F_2_ individuals had a mean (SD) fin melanophore count of 70.00 (23.63). With 45.16 (19.76) mean (SD) number of melanophores on the fin, the backcross (*M. zebra* (♀)×*M. benetos* (♂) backcross to *M. benetos*) began to resemble *M. benetos* which had a mean (SD) fin melanophore count of 19.73 (9.95) (Fig. 4).

The ANOVA results indicated that both scale (*F* = 149.913, *d.f.* = 5, *p* = 2e^−16^) and fin (*F* = 118.772, *d.f.* = 5, *p* = 2e^−16^) melanophore densities were significantly different among parental, F_1_, F_2_ and backcross generations. Tukey's post-hoc test revealed that melanophore counts in both scale (*p*<1e^−10^) and fin (*p*<1e^−10^) were much higher in *M. zebra* than that in *M. benetos*. Hybrid individuals had intermediate melanophore counts on both tissue types (Table 1, 2). For scale melanophore counts, Tukey's multiple comparisons suggested that *M. zebra* was slightly, though significantly, different from the backcross with *M. zebra* (*p* = 0.045), while no significant difference existed between *M. benetos* and the backcross with *M. benetos* (*p* = 0.083). Further, no statistically significant difference was found between the F_1_ and F_2_ generations in scale melanophore count (*p = *0.977). For the melanophore count in fin tissues, *M. zebra* fin melanophore counts were significantly greater than the backcross with *M. zebra* (*p*<1e^−10^). Likewise, *M. benetos* had significantly fewer fin melanophores than the backcross to *M. benetos* (*p*<1e^−10^). Significant differences between F_1_ and F_2_ generations in melanophore count in fin was observed as well (*p*<1e^−5^).

**Table 1:** The square root transformed means (standard errors) of melanophore counts (within a 0.25 mm^2^ area for scales (Fig. 1).

Scale		Model		
		Expected Additive	Expected Additive-Dominance	
	Observed			
*M. zebra*	8.84 (0.12)	9.01 (0.10)	9.03 (0.11)	
*M. benetos*	3.97 (0.21)	3.63 (0.11)	3.67 (0.16)	
F_1_	6.22 (0.12)	6.32 (0.05)	6.30 (0.10)	
F_2_	6.31 (0.07)	6.32 (0.05)	6.32 (0.05)	
Backcross to *M. zebra*	8.23 (0.15)	7.67 (0.06)	7.66 (0.06)	
Backcross to *M. benetos*	4.78 (0.13)	4.98 (0.07)	4.98 (0.07)	
		**χ^2^_2_ = 22.10, ** ***p*** **<0.0001**	**χ^2^_1_ = 22.19, ** ***p*** **<0.0001**	**Λ_1_ = 0.09, ** ***p*** ** = 0.76**

Given are empirical counts, expected values assuming an additive genetic model, and expected values assuming an additive-dominance model. χ^2^ tests were used to compare the genetic models to the observed data. A likelihood-ratio test was used to test if the additive-dominance model better explained the data than the additive model (Λ).

**Table 2:** The square root transformed means (standard errors) of melanophore counts (within a 0.25 mm^2^ area for pelvic fins (Fig. 1).

Fin		Model		
		Expected Additive	Expected Additive-Dominance	
	Observed			
*M. zebra*	10.42 (0.16)	11.00 (0.13)	10.25 (0.15)	
*M. benetos*	4.30 (0.17)	5.13 (0.13)	4.35 (0.16)	
F_1_	9.17 (0.16)	8.07 (0.07)	9.06 (0.12)	
F_2_	8.25 (0.09)	8.07 (0.07)	8.18 (0.06)	
Backcross to *M. zebra*	8.66 (0.25)	9.53 (0.08)	9.65 (0.08)	
Backcross to *M. benetos*	6.55 (0.35)	6.59 (0.09)	6.70 (0.09)	
		**χ^2^_2_ = 18.58, ** ***p*** **<0.001**	**χ^2^_1_ = 101.33, ** ***p*** **<0.0001**	**Λ_1_ = 82.74, ** ***p*** **<0.0001**

Given are empirical counts, expected values assuming an additive genetic model, and expected values assuming an additive-dominance model. χ^2^ tests were used to compare the genetic models to the observed data. A likelihood-ratio test was used to test if the additive-dominance model better explained the data than the additive model (Λ).

### Quantitative genetic analysis of color pattern

Neither the additive model (Table 1; χ^2^_1_ = 22.19, *p* = 0.00018) nor the additive-dominance model (Table 1; χ^2^_2_ = 22.10, *p* = 6.22e^−5^) explained the pattern of melanophore inheritance on scales. Furthermore, the additive-dominance model did not significantly improve the fit of the model to the observed data (Table 1; Λ_1_ = 0.09, *p* = 0.76). The inheritance of melanophore in the pelvic fin tissues showed a similar pattern: both the additive model (Table 2; χ^2^_1_ = 101.33, *p*<1e^−10^) and additive-dominance model were rejected (Table 2; χ^2^_2_ = 18.58, *p* = 0.0003). However, the additive-dominance model did significantly improve the fit of the model to the observed data (Table 2; Λ_1_ = 82.74, *p*<1e^−10^). The rejection of the additive and additive-dominance models suggests the action of epistasis, which was supported for both the scale (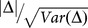  = 0.019<1.96) and fin (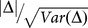  = 0.16<1.96) phenotypes. Genetic factor analysis suggested that a large number of genetic factors control melanophore patterning of the scales and pelvic fins. The estimated minimum number of genetic factors ranged from −5 (SD = 11.02) to −30 (SD = 55.09) for scale and 29 (SD = 229.27) to 141 (SD = 1496.37) for the pelvic fin depending on the size of allelic effects (Table 3).

**Table 3:** The estimated effective number of factors (standard deviation) influencing scale and fin melanophore counts for a range of allelic effects (C_a_ = 0 assumes the equivalence of allelic effects; C_a_ =  assumes a normal distribution of allelic effects; C_a_ = 1 assumes the allelic effects have a negative exponential distribution; C_a_ = 4 assumes that allelic effects have a leptokurtic distribution).

C_a_	Scale n_e_		Fin n_e_	
0	−5.33	(11.02)	29.02	(299.27)
0.25	−6.92	(13.77)	36.02	(374.09)
1	−11.67	(22.03)	57.03	(598.55)
4	−30.69	(55.09)	141.08	(1496.37)

### Test for association between male courting effort and female mate choice

**Figure 5:**
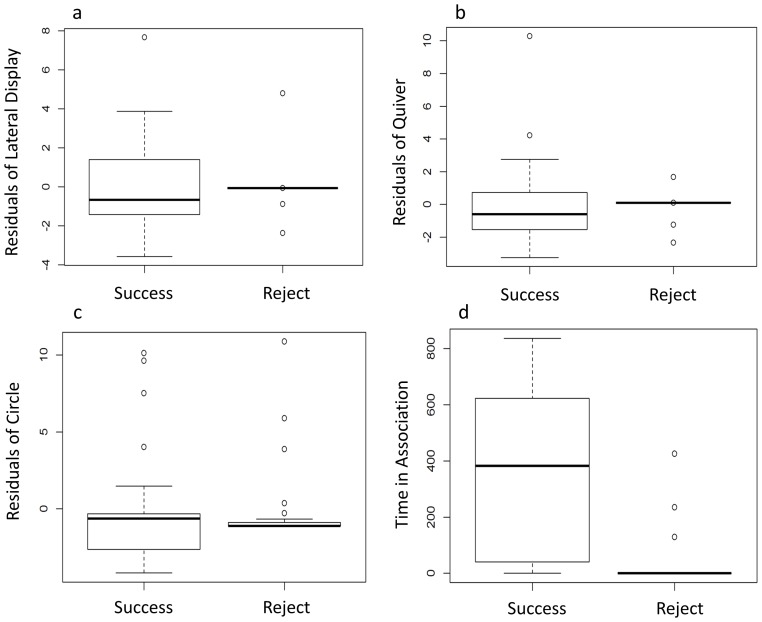
Comparisons of the residuals derived from the regression of male lateral displays (a), quivers (b), and circles (c) against the amount of time that female stayed in each side of the tank between the successfully mated males and rejected males; further comparison of the time in association (d) that female spent with the successful mated males and rejected males is shown.

No statistically significant effect of male courtship effort on female preference was observed for any of the three male behaviors: lateral display (*p* = 0.17, Fig. 5a), quiver (*p* = 0.20, Fig. 5b) and circle (*p* = 0.89, Fig. 5c). However, females did spend significantly more time associating with males they eventually mated with compared to the males that were rejected: in 21 of 24 trials the female spent more time with the preferred male (*p* = 1.82e^−6^, Fig. 5d).

### Quantitative genetic analysis of female mate choice

**Figure 6:**
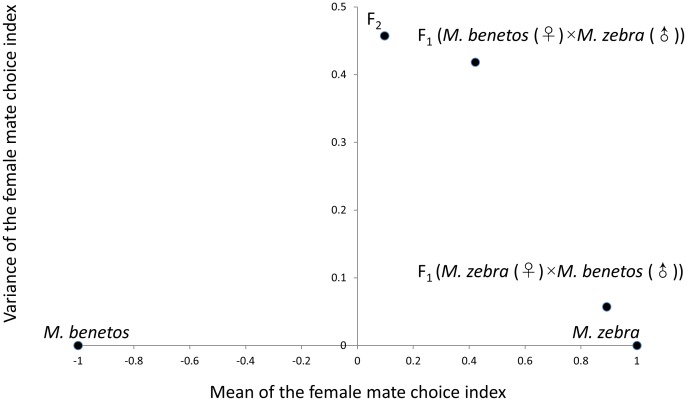
Mean (x axis) and variance (y axis) of the female mate choice index in the parentals, and F_1_ and F_2_ hybrids.

The mean (SD) female mate choice index for *M. zebra* and *M. benetos* was 1(0) and −1(0), respectively and all hybrid crosses had intermediate means with varying amounts of dominance in the direction of *M. zebra* (Fig. 6). The mean (SD) mate choice for the 17 F_1_ females derived from the cross between *M. benetos* (♀)×*M. zebra* (♂) equaled 0.42 (0.64) and was significantly greater than 0 (i.e. there was significant dominance in the direction of *M. zebra*) (*t* = 2.6887, *d.f.* = 16, *p* = 0.008). The mean (SD) of mate choice for the 12 F_1_ hybrid derived from the cross between *M. zebra* (♀)×*M. benetos* (♂) was 0.89 (0.24) and was significantly greater than 0 (*t* = 12.93, *d.f.* = 11, *p* = 2.687e^−8^). The Welch two sample *t*-test showed a significant difference between the two reciprocal crosses (*t* = 2.74, *d.f*. = 21.61, *p* = 0.023). As only one direction of the cross (*M. zebra* (♀)×*M. benetos* (♂)) could be used to make F_2_ hybrids, genetic factor analyses of female mate choice, which requires F_1_ data, utilized only the F_1_ data from this cross. The mean of the mate choice index for F_2_ hybrids was 0.097 (0.68) and was significantly greater than 0 (*t* = 1.9387, *d.f.* = 181, *p* = 0.027) (Fig. 6). The number of F_2_ hybrids that mated exclusively with *M. zebra* or *M. benetos* was 39 and 31, respectively. One hundred twelve F_2_ females mated with both *M. zebra* and *M. benetos* (Table 4). This pattern of female mate preference in the F_2_ significantly deviated from random mating (χ^2^ = 65.68, *d.f.* = 2, *p*<0.0001). The minimum number of genetic factors affecting female mate choice was 1.16 (0.02). After Zeng's correction [56] as described in Lynch and Walsh [55] was applied, the estimated number of genetic factors ranged from 1.2 to 1.9, depending on the distribution of allelic effects (Table 5).

**Table 4:** Results of the female mate choice assay.

	*M. zebra*	*M. benetos*	*M. zebra & M. benetos*	Total (Mean of the mate choice index)
*M. zebra*	10	0	0	10(1)
*M. benetos*	0	10	0	10 (−1)
F_1_ (*M. benetos* (♀)×*M. zebra* (♂))	7	0	10	17 (0.42)
F_1_ (*M. zebra* (♀)×*M. benetos* (♂))	9	0	3	12 (0.89)
F_2_	39	31	112	182 (0.097)

Females of *M. zebra*, *M. benetos*, F_1_ and F_2_ were tested in a two way mate choice tank (Fig. 2), The numbers of individuals tested for female mate choice and the mean of the mate choice in each generation are given.

**Table 5:** The estimated effective number of factors influencing cichlid female mate choice for a range of allelic effects (C_a_ = 0 assumes the equivalence of allelic effects; C_a_ = 0.25 assumes a normal distribution of allelic effects; C_a_ = 1 assumes the allelic effects have a negative exponential distribution; C_a_ = 4 assumes that allelic effects have a leptokurtic distribution).

C_a_	n	Standard deviation
0	1.2	0.02
0.25	1.2	0.04
1	1.3	0.09
4	1.9	0.57

### Test for phenotypic correlation between female mate choice and male color pattern

**Figure 7:**
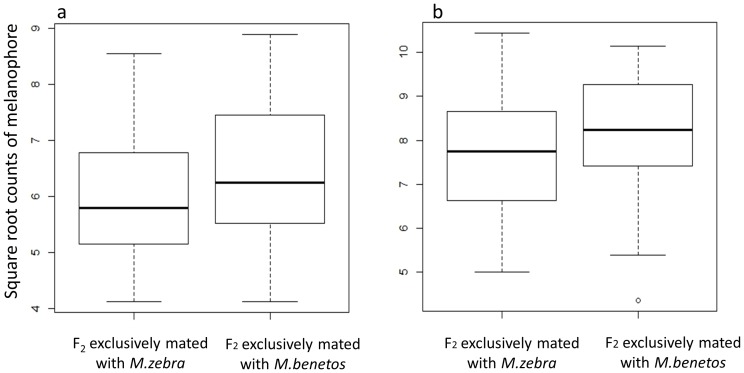
Comparison of F_2_ scale (a) and fin (b) phenotypes between individuals that exclusively mated with *M. zebra* or *M. benetos*.

When the melanophore counts of those F_2_ that mated exclusively with *M. benetos* were compared to the melanophore counts of those F_2_ that mated exclusively with *M. zebra* no significant differences were observed in either the scale (*F* = 1.4727, *d.f*. = 1, *p* = 0.2296) or fin data (*F* = 0, *d.f.* = 1, *p* = 0.9999) (Fig. 7a, b).

## Discussion

### Genetic factors influencing melanistic patterning

The genetic architecture of sexually selected traits remains poorly understood. To gain a better understanding of the number of genetic factors involved, the modes of gene action, and the genetic correlation between male signaling and female mating preference phenotypes, we conducted a biometric analysis of an East African cichlid pair. Our results suggest that many loci are involved in melanistic patterns of scales and pelvic fins. This is indicated by the large number of genetic factors that was estimated for fin melanophore counts and the negative number of genetic factors for scale melanophores. The estimated negative numbers of factors may be a result of many genes of small effect contributing to a phenotype [55] or violations of the assumptions of the Castle-Wright method. The Castle-Wright method assumes that alleles are alternatively fixed in two parental lines and have equal allelic effects [55]. However, our results clearly suggest that genes with partially dominant effects underlie the melanistic patterning and female mate choice. In addition, a relatively small sample size for the backcross lines can affect the estimates as well [55]. Therefore, the estimated number of genetic factors should only be considered as a lower bound of the genetic factors underlying these traits.

Our finding differs from previous investigations of the genetic architecture of coloration in East African cichlids. Using a similar biometric approach, Barson et al. [58] found that blue body coloration of cichlids was influenced by 4–7 genes, Magalhaes and Seehausen [45] found that 2–4 genes appear to determine red coloration in a hybrid cross of Lake Victoria cichlids, and O'Quin et al. [43] found that 1–4 genes control the pigmentation differences between a pair of Lake Malawi cichlids one of which, *M. zebra*, is also a focal species of this study. Additional efforts by O′Quin et al. (2013) identified one QTL associated with pelvic fin melanophore pigmentation but failed to identify any significant QTL for melanophore counts in scales and a number of additional tissue types including the caudal fin and cheek. The authors suggested that multiple genes with small effects were underlying the number of melanophores in these areas of the body which is consistent with our findings. A recent mapping study identified 41 QTL and 13 epistatic interactions were underlying the melanistic patterning in cichlids [59]. All of these studies consistently demonstrated that the additive genetic model is inadequate for explaining the observed phenotypic variance. In our experiment, both the additive and the additive-dominance models of inheritance were rejected when examining the distribution of melanistic phenotypes in the hybrid crosses, while the presence of epistatic interactions was supported.

### Male courting effort versus female mate choice

Parental investment is largely asymmetrical in haplochromine cichlids, including the vast majority of Lake Malawi cichlids: females are promiscuous mouth brooders which produce relatively large, energetically expensive eggs that are fertilized by males that contribute little other than sperm [60]. This asymmetrical investment in parental care is thought to have led to the evolution of strong female mate choice [61] and vigorous male courtship [62] in this system [9], [63], [64].

In our mate choice trials, females were allowed to interact with males. As a result, females that stayed longer in one side of the arena tended to elicit more male courting efforts. Many studies suggest that vigorous male courting effort can significantly affect female mate choice [65]–[67]. In our study, by examining the effect of male courtship effort via statistically removing the effect of time spent with a female, we found that none of the three male courting behaviors significantly influenced female mate choice. Consistent with Haesler and Seehausen [29], these results suggest that female mate choice probably occurs prior to the onset of intense courtship [50]. Alternatively, male courtship displays may have an indirect effect on reproductive success or may be maintained through pleiotropic effects. Nonetheless, we did find a significant difference between the times that female spent with successful males compared to the rejected males. Time spent in association may be a good indicator of female preference particularly in interspecific comparisons. However this measure lacks the necessary accuracy when examining individual preferences within F_2_ populations.

### Segregation of female mate choice among F_2_ female hybrids

Quantifying the genetic basis of female mate choice can improve our understanding of reproductive isolation and speciation. In our study, females of the parental species, *M. zebra* and *M. benetos*, mated exclusively with conspecific males which is consistent with the previous study of these species [28].

Despite significant differences in the mate choice of the reciprocal hybrid F_1_ females, F_1_ hybrids derived from both directions exhibited a preference for *M. zebra* males and an additive genetic effect in the F_1_ was rejected. Likewise, the F_2_ females exhibited a dominant preference for the *M. zebra* phenotype; however, the degree of dominance in the F_2_ was much smaller than in either of the F_1_ lines. Why the degree of dominance of mate choice diminished in the F_2_ is unclear. It may be the result of a ‘parent of origin’ epigenetic phenomenon [68]–[71] or masking effects by maternal imprinting on an intermediate F_1_ phenotype in the F_2_ [72] (early imprinting would not have occurred in the F_1_ as these were the only lines raised entirely outside of a female's buccal cavity and would have escaped any developmental imprinting). Nonetheless, female mate choice significantly deviates from random mating in the F_2_ population indicating that female mate choice has a strong heritable component. In addition, it is clear that the alleles influencing female mate choice in the two parental species have segregated in the F_2_ population to recapitulate the parental phenotypes. The strong genetic component of female mate choice is further corroborated by our behavioral experiments in which no statistical correlation between male courting effort and female mate choice was observed.

Future studies should further explore the dominance of *M. zebra* mate preferences. Such additional studies could provide valuable insight into the diversification of Lake Malawi's cichlids. Directional selection is believed to drive the evolution of dominance [73]–[75] and the dominance of *M. zebra* mate preferences may reflect ongoing directional selection operating on mate preferences. In addition, dominance could affect the tempo of genetic isolation of diverging lineages as a partially dominant allele will increase in frequency faster than a recessive allele [76]. Furthermore, the pattern of dominance biased preference for *M. zebra* would lead to biased introgression into the *M. zebra* gene pool. Lastly, the observed dominance, if confirmed, may be, one of the few documented empirical examples of “Haldane's sieve” [77] in female mate choice. “Haldane's sieve” predicts that if species-level sexual isolation results primarily from directional selection, then interspecific hybrid females should discriminate against males of either or both parental types [73]. It is also worth noting that only female hybrids were produced from the 11 *M. benetos* (♀)×*M. zebra* (♂) F_1_ broods in our attempt to make the reciprocal cross. This might suggest that postzygotic isolation has started to evolve between the studied species. If further experiments corroborate our observation, this might be among the first experimental evidence for Haldane's rule in cichlids [78].

### Genetic factors influencing female mate choice

Female mate choice is thought to accelerate the rate of species divergence [79]. Quantitative genetic models of mate choice [38], [39], [80] and empirical studies [37], [40], [81] have suggested that the number of genes contributing to a phenotype influences the pace of phenotypic evolution. As such, quantifying the genetic elements influencing female mating preference in East African cichlids may provide a genetic mechanism which may have contributed to the extraordinary speed of this species radiation. In our study, we found that female mate choice is controlled by few genetic factors. Our result is consistent with an empirical study of a pair of Lake Victoria cichlids [29] and suggests that the rapid diversification of East African cichlids may have been facilitated by few genetic factors underlying female mate choice.

### Correlation between female mate choice and color pattern

In a previous study, visual cues, and melanistic markings in particular, were sufficient for female mate choice in this species pair [28]. Furthermore, quantitative genetic models and empirical studies suggest that a physical linkage of female mate choice and male secondary sexual traits likely accelerates speciation in cichlids [3], [30], [37], [82]. However in this study, female mate choice and melanistic color pattern were not associated with each other in F_2_ females and there was no evidence of linkage between the male signal and female mate preferences. However, as noted by Van der Sluijs et al. [48], if many genes underlie both female mate choice and male secondary traits, it may be difficult to detect weakly linked genes. Nonetheless, our results suggest that factors other than the physical linkage of melanistic patterns and mate choice have contributed to the divergence of this species pair. Future experiments are needed to examine the role of physical linkage of genes contributing to preferred male phenotypes and female mate preferences in the broader East African cichlid radiation.

## Conclusions

The genetic mechanisms underlying the extraordinary East African cichlids radiation still remain elusive. In this study, using a pair of closely related, yet reproductively isolated Malawian cichlids as the model, we investigated the genetic architecture of two evolutionary significant traits, male color pattern and female mate choice, with quantitative genetic analyses. Our results suggest that melanistic color patterns are influenced by many non-additively acting genetic factors, while female mate choice may be controlled by a few non-additive genetic factors. Female mate choice is a heritable trait and male courting effort had little influence on it. Furthermore, our joint analysis of color pattern and female mate choice suggests that the genes underlying these two traits are unlikely to be physically linked. Cichlids reproductive isolation may evolve rapidly owing to the few genetic factors underlying the female mate choice. The genetic nature of male color pattern is even more complex, multiple loci with dominant and epistatic interaction were involved in its formation. Further studies are needed to identify the loci underlying female mate choice and male color pattern to understand the role these evolutionary significant traits played in cichlid speciation.

## Supporting Information

S1 Video **Female mate choice assay tank.**
(WMV)

## References

[1] Chenoweth SF, McGuigan K (2010) The genetic basis of sexually selected variation. Annual Review of Ecology, Evolution, and Systematics 41:81–101.

[2] Hansen TF (2006) The evolution of genetic architecture. Annual Review of Ecology, Evolution, and Systematics 37:123–157.

[3] Butlin RK, Ritchie MG (1989) Genetic coupling in mate recognition systems: what is the evidence? Biological Journal of the Linnean Society 37:237–246.

[4] Trexler JC (1990) Genetic architecture of behavior in fishes and the response to selection. Ann Zool Fennici 27:149–163.

[5] Danley PD, Husemann M, Ding B, DiPietro LM, Beverly EJ, et al (2012) The impact of the geologic history and paleoclimate on the diversification of East African cichlids. International Journal of Evolutionary Biology 2012:20. 10.1155/2012/574851 PMC3408716 22888465

[6] Turner GF, Seehausen O, Knight ME, Allender CJ, Robinson RL (2001) How many species of cichlid fishes are there in African lakes? Molecular Ecology 10:793–806. 11298988 10.1046/j.1365-294x.2001.01200.x

[7] Koblmüller S, Sefc K, Sturmbauer C (2008) The Lake Tanganyika cichlid species assemblage: recent advances in molecular phylogenetics. Hydrobiologia 615:5–20.

[8] Dominey WD (1984) Effects of sexual selection and life history on speciation: species flocks in African cichlids and Hawaiian *Drosophila*. In Evolution of fish species flocks Echelle AAKornfield Ieditors. Orono, ME: University of Maine Press.

[9] Kocher TD (2004) Adaptive evolution and explosive speciation: the cichlid fish model. Nat Rev Genet 5:288–298. 15131652 10.1038/nrg1316

[10] Turner GF, Burrows MT (1995) A model of sympatric speciation by sexual selection. Proceedings of the Royal Society of London Series B: Biological Sciences 260:287–292.

[11] Streelman JT, Albertson RC, Thomas DK (2003) Genome mapping of the orange blotch colour pattern in cichlid fishes. Molecular Ecology 12:2465–2471. 12919484 10.1046/j.1365-294x.2003.01920.x

[12] Roberts RB, Ser JR, Kocher TD (2009) Sexual conflict resolved by invasion of a novel sex determiner in Lake Malawi cichlid fishes. Science 326:998–1001. 19797625 10.1126/science.1174705 PMC3174268

[13] O'Quin C, Drilea A, Conte M, Kocher T (2013) Mapping of pigmentation QTL on an anchored genome assembly of the cichlid fish, *Metriaclima zebra* . BMC Genomics 14:287. 23622422 10.1186/1471-2164-14-287 PMC3691601

[14] Genner MJ, Turner GF (2012) Ancient hybridization and phenotypic novelty within Lake Malawi's cichlid fish radiation. Molecular Biology and Evolution 29:195–206. 22114359 10.1093/molbev/msr183

[15] Konings A (2007) Malawi cichlids in their natural habitat 4^th^ Edition. Cichlid Press. El Paso, Texas, USA.

[16] Seehausen Mayhew, Alphen JJMV (1999) Evolution of colour patterns in East African cichlid fish. Journal of evolutionary biology 12:514–534.

[17] Kelley JL, Fitzpatrick JL, Merilaita S (2013) Spots and stripes: ecology and colour pattern evolution in butterflyfishes. Proceedings of the Royal Society B: Biological Sciences 280. 10.1098/rspb.2012.2730 PMC3619473 23427170

[18] Husemann M, Tobler M, McCauley C, Ding B, Danley PD (2014) Evolution of body shape in differently coloured sympatric congeners and allopatric populations of Lake Malawi's rock-dwelling cichlids. Journal of Evolutionary Biology: 826–839. 24617299 10.1111/jeb.12353

[19] Ding B, Curole J, Husemann M, Danley PD (2014) Habitat complexity predicts the community diversity of rock-dwelling cichlid fish in Lake Malawi, East Africa. Hydrobiologia.

[20] Danley PD (2011) Aggression in closely related Malawi cichlids varies inversely with habitat complexity. Environmental Biology of Fishes 92:275–284.

[21] Dijkstra PD, Seehausen O, Pierotti MER, Groothuis TGG (2007) Male–male competition and speciation: aggression bias towards differently coloured rivals varies between stages of speciation in a Lake Victoria cichlid species complex. Journal of Evolutionary Biology 20:496–502. 17305815 10.1111/j.1420-9101.2006.01266.x

[22] Dijkstra PD, Hemelrijk C, Seehausen O, Groothuis TGG (2009) Color polymorphism and intrasexual competition in assemblages of cichlid fish. Behavioral Ecology 20:138–144.

[23] Hert E (1991) Female choice based on egg-spots in *Pseudotropheus aurora* Burgess 1976, a rock-dwelling cichlid of Lake Malaŵi, Africa. Journal of Fish Biology 38:951–953.

[24] Seehausen O, Terai Y, Magalhaes IS, Carleton KL, Mrosso HDJ, et al (2008) Speciation through sensory drive in cichlid fish. Nature 455:620–626. 18833272 10.1038/nature07285

[25] Seehausen O, van Alphen JJM (1998) The effect of male coloration on female mate choice in closely related Lake Victoria cichlids (*Haplochromis nyererei* complex). Behavioral Ecology and Sociobiology 42:1–8.

[26] Knight ME, Turner GF, Rico C, Van Oppen MJH, Hewitt GM (1998) Microsatellite paternity analysis on captive Lake Malawi cichlids supports reproductive isolation by direct mate choice. Molecular Ecology 7:1605–1610.

[27] Couldridge VCK, Alexander GJ (2002) Color patterns and species recognition in four closely related species of Lake Malawi cichlid. Behavioral Ecology 13:59–64.

[28] Kidd MR, Danley PD, Kocher TD (2006) A direct assay of female choice in cichlids: all the eggs in one basket. Journal of Fish Biology 68:373–384.

[29] Haesler MP, Seehausen O (2005) Inheritance of female mating preference in a sympatric sibling species pair of Lake Victoria cichlids: implications for speciation. Proceedings of the Royal Society B: Biological Sciences 272:237–245. 15705547 10.1098/rspb.2004.2946 PMC1634962

[30] Shaw KL, Lesnick SC (2009) Genomic linkage of male song and female acoustic preference QTL underlying a rapid species radiation. Proceedings of the National Academy of Sciences 106:9737–9742. 10.1073/pnas.0900229106 PMC2701026 19487670

[31] Lande R, Seehausen O, van Alphen JJM (2001) Mechanisms of rapid sympatric speciation by sex reversal and sexual selection in cichlid fish. Genetica 112:435–443. 11838780

[32] van Doorn GS, Noest AJ, Hogeweg P (1998) Sympatric speciation and extinction driven by environment dependent sexual selection. Proceedings of the Royal Society of London Series B: Biological Sciences 265:1915–1919.

[33] Higashi M, Takimoto G, Yamamura N (1999) Sympatric speciation by sexual selection. Nature 402:523–526. 10591210 10.1038/990087

[34] Panhuis TM, Butlin R, Zuk M, Tregenza T (2001) Sexual selection and speciation. Trends in Ecology & Evolution 16:364–371. 11403869 10.1016/s0169-5347(01)02160-7

[35] Merrill RM, Van Schooten B, Scott JA, Jiggins CD (2011) Pervasive genetic associations between traits causing reproductive isolation in *Heliconius* butterflies. Proceedings of the Royal Society B: Biological Sciences 278:511–518. 20810445 10.1098/rspb.2010.1493 PMC3025683

[36] Ritchie MG (2007) Sexual selection and speciation. Annual Review of Ecology, Evolution, and Systematics 38:79–102.

[37] Kronforst MR, Young LG, Kapan DD, McNeely C, O'Neill RJ, et al (2006) Linkage of butterfly mate preference and wing color preference cue at the genomic location of wingless. Proceedings of the National Academy of Sciences 103:6575–6580. 10.1073/pnas.0509685103 PMC1458925 16611733

[38] Lande R (1981) Models of speciation by sexual selection on polygenic traits. Proceedings of the National Academy of Sciences 78:3721–3725. 10.1073/pnas.78.6.3721 PMC319643 16593036

[39] Lande R (1982) Rapid origin of sexual isolation and character divergence in a cline. Evolution 36:213–223. 28563171 10.1111/j.1558-5646.1982.tb05034.x

[40] Moehring AJ, Llopart A, Elwyn S, Coyne JA, Mackay TFC (2006) The genetic basis of prezygotic reproductive isolation between *Drosophila santomea* and *D. yakuba* due to mating preference. Genetics 173:215–223. 16510787 10.1534/genetics.105.052993 PMC1461457

[41] Velthuis B-J, Yang W, Van Opijnen T, Werren JH (2005) Genetics of female mate discrimination of heterospecific males in *Nasonia* (Hymenoptera, Pteromalidae). Animal Behaviour 69:1107–1120.

[42] Takahashi T, Sota T, Hori M (2013) Genetic basis of male colour dimorphism in a Lake Tanganyika cichlid fish. Molecular Ecology 22:3049–3060. 23176589 10.1111/mec.12120

[43] O'Quin CT, Drilea AC, Roberts RB, Kocher TD (2012) A small number of genes underlie male pigmentation traits in Lake Malawi cichlid fishes. Journal of Experimental Zoology Part B: Molecular and Developmental Evolution 318:199–208. 10.1002/jez.b.22006 22544717

[44] Svensson O, Egger B, Gricar B, Woodhouse K, van Oosterhout C, et al (2011) Segregation of species-specific male attractiveness in F2 hybrid Lake Malawi cichlid fish. International Journal of Evolutionary Biology 2011. 10.4061/2011/426179 PMC3119475 21716739

[45] Magalhaes IS, Seehausen O (2010) Genetics of male nuptial colour divergence between sympatric sister species of a Lake Victoria cichlid fish. Journal of evolutionary biology 23:914–924. 20345823 10.1111/j.1420-9101.2010.01960.x

[46] Chenoweth SF, Blows MW (2006) Dissecting the complex genetic basis of mate choice. Nat Rev Genet 7:681–692. 16921346 10.1038/nrg1924

[47] Wagner WE (1998) Measuring female mating preferences. Animal Behaviour 55:1029–1042. 9632487 10.1006/anbe.1997.0635

[48] Van der Sluijs I, Seehausen O, Van Dooren TJM, Van Alphen JJM (2010) No evidence for a genetic association between female mating preference and male secondary sexual trait in a Lake Victoria cichlid fish. Current Zoology 56:57–64.

[49] Ohta T (1974) Movement of pigment granules within melanophores of an isolated fish scale. Effects of cytochalasin B on melanophores. Biological Bulletin 146:258–266. 4822764 10.2307/1540622

[50] McElroy DM, Kornfield I (1990) Sexual selection, reproductive behavior, and speciation in the mbuna species flock of Lake Malawi (Pisces: Cichlidae). Environmental Biology of Fishes 28:273–284.

[51] Van Staaden MJ, Smith AR (2011) Cutting the Gordian knot: Complex signaling in African cichlids is more than multimodal. Current Zoology 57:237–252.

[52] Plenderleith M, Oosterhout Cv, Robinson RL, Turner GF (2005) Female preference for conspecific males based on olfactory cues in a Lake Malawi cichlid fish. Biology Letters 1:411–414. 17148220 10.1098/rsbl.2005.0355 PMC1626363

[53] Baerends GP, Baerends-van Roon JM (1950) An introduction to the study of the ethology of the cichlid fishes. Behaviour Supplement: III-243.

[54] Hayman BI (1960) Maximum likelihood estimation of genetic components of variation. Biometrics 16:369–381.

[55] Lynch M, Walsh B (1998) Genetics and analysis of quantitative traits: Sinauer.

[56] Zeng Z (1992) Correcting the bias of Wright estimates of the number of genes affecting a quantitative character - a further improved method. Genetics 131:987–1001. 1325390 10.1093/genetics/131.4.987 PMC1205108

[57] R Development Core Team (2012) R: A language and environment for statistical computing. Vienna, Austria: R Foundation for Statistical Computing. pp. Retrieved from http://www.R-project.org.

[58] Barson N, Knight M, Turner G (2007) The genetic architecture of male colour differences between a sympatric Lake Malawi cichlid species pair. Journal of Evolutionary Biology 20:45–53. 17209998 10.1111/j.1420-9101.2006.01228.x

[59] Craig Albertson R, Powder KE, Hu Y, Coyle KP, Roberts RB, et al. (2014) Genetic basis of continuous variation in the levels and modular inheritance of pigmentation in cichlid fishes. Molecular Ecology: n/a-n/a. 10.1111/mec.12900 PMC4238941 25156298

[60] Parker A, Kornfield I (1996) Polygynandry in *Pseudotropheus zebra*, a cichlid fish from Lake Malawi. Environmental Biology of Fishes 47:345–352.

[61] Kuwamura T (1986) Parental care and mating systems of cichlid fishes in Lake Tanganyika: a preliminary field survey. Journal of Ethology 4:129–146.

[62] Gonzalez-Voyer A, Fitzpatrick JL, Kolm N (2008) Sexual selection determines parental care patterns in cichlid fishes. Evolution 62:2015–2026. 18489718 10.1111/j.1558-5646.2008.00426.x

[63] Sefc KM (2011) Mating and parental care in Lake Tanganyika's cichlids. International Journal of Evolutionary Biology 2011. 10.4061/2011/470875 PMC3142683 21822482

[64] Goldschmidt T, Witte F (1990) Reproductive strategies of zooplanktivorous Haplochromine cichlids (Pisces) from Lake Victoria before the Nile perch boom. Oikos 58:356–368.

[65] Kidd M, Dijkstra P, Alcott C, Lavee D, Ma J, et al (2013) Prostaglandin F2α facilitates female mating behavior based on male performance. Behavioral Ecology and Sociobiology 67:1307–1315.

[66] Steinwender B, Koblmüller S, Sefc K (2012) Concordant female mate preferences in the cichlid fish *Tropheus moorii* . Hydrobiologia 682:121–130. 10.1007/s10750-011-0766-5 PMC3841713 24293682

[67] Byers J, Hebets E, Podos J (2010) Female mate choice based upon male motor performance. Animal Behaviour 79:771–778.

[68] Bonduriansky R, Day T (2013) Nongenetic inheritance and the evolution of costly female preference. Journal of Evolutionary Biology 26:76–87. 23163399 10.1111/jeb.12028

[69] Pfennig DW, Servedio MR (2013) The role of transgenerational epigenetic inheritance in diversification and speciation. Non-Genetic Inheritance: 17–26.

[70] Garfield AS, Cowley M, Smith FM, Moorwood K, Stewart-Cox JE, et al (2011) Distinct physiological and behavioural functions for parental alleles of imprinted Grb10. Nature 469:534–538. 21270893 10.1038/nature09651 PMC3031026

[71] Mott R, Yuan W, Kaisaki P, Gan X, Cleak J, et al (2014) The architecture of Parent-of-Origin effects in mice. Cell 156:332–342. 24439386 10.1016/j.cell.2013.11.043 PMC3898482

[72] Verzijden MN, ten Cate C (2007) Early learning influences species assortative mating preferences in Lake Victoria cichlid fish. Biology Letters 3:134–136. 17287180 10.1098/rsbl.2006.0601 PMC2375935

[73] Noor MAF (1999) Reinforcement and other consequences of sympatry. Heredity 83:503–508. 10620021 10.1038/sj.hdy.6886320

[74] Barton NH, Keightley PD (2002) Understanding quantitative genetic variation. Nat Rev Genet 3:11–21. 11823787 10.1038/nrg700

[75] Broadhurst PL (1979) The experimental approach to behavioral evolution. In: Royce JR, Mos LPeditors. Theoretical Advances in Behavior Genetics. Alphen aan den Rijn, The Netherlands: Sijthoff & Noordhoff. pp.43–100.

[76] Falconer DS, Mackay TFC (1996) Introduction to quantitative genetics. New York: Longman.

[77] Orr HA, Betancourt AJ (2001) Haldane's sieve and adaptation from the standing genetic variation. Genetics 157:875–884. 11157004 10.1093/genetics/157.2.875 PMC1461537

[78] Stelkens RB, Young KA, Seehausen O (2010) The accumulation of reproductive incompatibilities in African cichlid fish. Evolution 64:617–633. 19796149 10.1111/j.1558-5646.2009.00849.x

[79] Seehausen O, van Alphen JJM, Witte F (1997) Cichlid fish diversity threatened by eutrophication that curbs sexual selection. Science 277:1808–1811.

[80] Rettelbach A, Hermisson J, Dieckmann U, Kopp M (2011) Effects of genetic architecture on the evolution of assortative mating under frequency-dependent disruptive selection. Theoretical Population Biology 79:82–96. 21192962 10.1016/j.tpb.2010.12.001

[81] Laturney M, Moehring AJ (2012) The genetic basis of female mate preference and species isolation in *Drosophila* . International Journal of Evolutionary Biology 2012:13. 10.1155/2012/328392 PMC3432541 22957299

[82] Safran RJ, Scordato ESC, Symes LB, Rodríguez RL, Mendelson TC (2013) Contributions of natural and sexual selection to the evolution of premating reproductive isolation: a research agenda. Trends in Ecology & Evolution 28:643–650. 24054911 10.1016/j.tree.2013.08.004

